# Patients with History of Metastasis Have Differing Surgical Indications and Increased Perioperative Risk Following Revision Total Joint Arthroplasty

**DOI:** 10.1177/21514593251366161

**Published:** 2025-08-06

**Authors:** Aroob Zaheer, Alexander S. Vo, Guillermo Ramirez Campos, Nithin Gupta, Morgan Gable, Zachary Jodoin, Tyler K. Williamson, Frank A. Buttacavoli

**Affiliations:** 1 483612 University of the Incarnate Word School of Osteopathic Medicine, San Antonio, TX, USA; 2 364432 Campbell University School of Osteopathic Medicine, Lillington, NC, USA; 3 Department of Orthopaedic Surgery, 14742 University of Texas Health Science Center at San Antonio, San Antonio, TX, USA

**Keywords:** total joint arthroplasty, revision, metastasis, metastatic disease, cancer, complications

## Abstract

**Introduction:**

Revision arthroplasty is an invasive procedure with increased morbidity relative to primary joint arthroplasty. Therefore, patients with metastatic cancer (Met) undergoing revision total joint arthroplasty (rTJA) may be at greater risk. This study assesses early postoperative outcomes among Met patients undergoing rTJA.

**Materials and Methods:**

We reviewed the National Surgical Quality Improvement Program (NSQIP) database from 2015 to 2020 to evaluate rTHA/rTKA with Met and Non-Met. Univariate analysis and multivariate logistic regression were used to evaluate associations of Met patients compared with outcomes using odds ratio (OR) and 95% confidence interval (CI). Discriminatory accuracy was assessed using Receiver operating characteristic (ROC) curve and quantified using C-statistic.

**Results:**

Adjusted analysis revealed Met patients undergoing rTKA were more likely to experience any complication (OR: 2.56, CI: [1.48–4.43]), major complication (OR: 2.17, CI: [1.24–3.82]), and mortality (OR: 7.99, CI: [2.70–23.65]). Met patients undergoing rTHA had higher associations with any complication (OR: 2.40, CI: [1.65–3.49]), major complication (OR: 2.19, CI: [1.47–3.25]), DVT (OR: 4.82, CI: [1.92–12.10]), and mortality (OR: 3.67, CI: [1.43–9.41]). Frailty had superior predictability of extended length of stay (C: 0.625 [0.619–0.630]) and mortality (C: 0.851 [0.824–0.880]).

**Conclusions:**

Patients with metastatic cancer have elevated risk of complications after revision arthroplasty but may have moderate predictability by frailty assessment. Surgeons can utilize this information to emphasize protective strategies to mitigate risk during and following total joint arthroplasty.

**Level of Evidence:**

III.

## Introduction

In the United States, total joint arthroplasty (TJA) has become one of the most common surgical procedures across all specialties, providing patients with greater mobility and an improved quality of life.^1,2^ Although advancements in technology and overall care have allowed for excellent outcomes in patients undergoing TJA, a subset of patients require revision due to surgical site infections, wound healing problems, aseptic loosening, and other postoperative complications.^3,4^ As the incidence of total joint arthroplasty rises, the demand for revision total joint arthroplasty (rTJA) concurrently rises and is expected to increase by 600% from 2005 to 2030.^5,6^

Studies have shown there to be greater complication rates, longer hospital stays, and increased prosthetic joint infections in patients undergoing revision TJA compared to primary cases.^7^ Furthermore, revision arthroplasty has become increasingly relevant in the context of the rising number of patients living with metastatic cancer. Over the past decade, cancer mortality rates have decreased annually by over 1%, resulting in approximately 13.7 million Americans currently living with a history of cancer.^8^ With certain cancers prone to metastasizing to bone, these patients may be at an additional increased risk for complications in an already complicated surgery.^9,10^ Decisions on surgical approaches are often tailored to a patient’s estimated survival risk. Therefore, understanding the relationship between metastatic cancer and rTJA can help improve surgical planning and patient management strategies.

The primary objective of this study is to assess the early postoperative outcomes in patients with metastatic cancer undergoing revision TJA. With this intent, we had four questions we sought to answer: (1) “Do patients with history of metastasis undergo revision TJA for the same indications as unaffected peers?”, (2) “Are patients with history of metastasis at higher risk for complications following revision total joint arthroplasty compared to unaffected peers?”, (3) “Are patients with history of metastasis at higher risk for complications during revision TKA or revision THA?”, and (4) “Is frailty more predictive of outcomes following revision TJA in patients with metastatic cancer?”. By focusing on this specific patient cohort, the information analyzed can be important in improving surgical management, postoperative care, and its implications for clinical practice. Given the challenges and outcomes associated with rTJA in patients with metastatic cancer, we hypothesize that postoperative complication rates will be higher in this population and more detailed medical profile assessments, like frailty, will provide better predictability of outcomes following revision TJA.

## Materials and Methods

The National Surgical Quality Improvement Program (NSQIP) database from 2015 to 2020 was queried for patients undergoing revision total hip arthroplasty (Current Procedural Terminology [CPT] codes 27134, 27137, 27138) and revision total knee arthroplasty (CPT codes 27486, 27487).^11^ The NSQIP is a database used to assess the quality of surgical care by collecting information on adverse events after surgery from participating sites across the nation. Due to the de-identification and public availability of the data, IRB approval was not required. Patients were included if undergoing one of the above procedures with an International Classification of Diseases (ICD)-9 or ICD-10 code related to indication for revision surgery.

### Population Characteristics and Variables

Baseline population characteristics were recorded and included age, sex, and race. Comorbidities included: Independent function, diabetes mellitus (T2DM), chronic obstructive pulmonary disorder (COPD), congestive heart failure (CHF), smoking status, dyspnea, hypertension (HTN), disseminated cancer (Met), chronic steroid use, chronic kidney disease stage 3 or greater (CKD), weight loss, body mass index (BMI), ASA grade, and bleeding disorders. The 5-factor modified frailty index (mFI-5) is a frailty index originally developed by Chimukangara et al. and validated using the NSQIP.^12^ The Risk Analysis Index (RAI) was originally described by Hall et al and was subsequently recalibrated using the NSQIP in order to validate it for use in non-veteran surgical patients, termed the RAI-Rev.^13,14^ It is calculated using 11 weighted variables. The frailty cutoffs used in this study were: ≤20 = Nonfrail, 21–30 = Prefrail, 31–40 = frail, and ≥41 = Severely Frail. Hospital admission variables included length of stay, operative time, preoperative hemoglobin, and discharge disposition. After implementing inclusion criteria, a total of 44 274 patients undergoing revision total joint arthroplasty were identified. There were 18 372 patients that underwent revision THA (41.5%) and 25 902 underwent revision TKA (58.5%). Of these, 223 patients had a history of disseminated cancer (Met). The demographics, surgical details, and disposition of each are compared between Met and Non-Met patients in Table 1. Patients with disseminated cancer had higher BMI, frailty, ASA, prevalence of CHF, and were less often smokers.

**Table 1.** Demographic and Surgical Details

Surgery	Met	Non-met	*P*-value
Age	67.9 ± 12.6	66.5 ± 11.4	.064
Sex (% women)	56.5%	56.6%	.973
Body mass index (kg/m^2^)	29.5 ± 6.6	32.1 ± 7.3	<.001
ASA classification	3.1 ± 0.6	2.7 ± 0.6	<.001
Smoking within one year of surgery	8.1%	12.3%	.056
Hypertension	57.4%	63.9%	.046
Congestive heart failure	3.6%	1.0%	<.001
Type 2 diabetes	16.6%	18.7%	.432
Chronic kidney disease	1.8%	0.8%	.106
Chronic obstructive pulmonary disease	6.3%	5.6%	.647
mFI-5	0.9 ± 0.9	0.9 ± 0.8	.942
RAI-rev	36.6 ± 4.4	21.7 ± 5.5	<.001
Revision THA	63.7%	41.4%	<.001
Revision TKA	36.3%	58.6%	<.001
Operative time (minutes; median [IQR])	143 [83-175]	137 [88-171]	.247
Length of stay (days)	7.1 ± 7.0	3.7 ± 4.4	<.001
Non-home discharge	39.5%	24.9%	<.001

### Outcomes and Complications

The primary outcome of this study was 30-day complication rates following revision total joint arthroplasty. Complications assessed included urinary tract infection, surgical site infection, sepsis, deep vein thrombosis, pulmonary embolism, cardiac complications (myocardial infarction and cardiac arrest), pulmonary complications (pneumonia, reintubation), as well as need for postoperative blood transfusion. Secondary outcomes included discharge disposition, unplanned readmission, unplanned reoperation, extended length of stay (eLOS) (>3 days), major complications and mortality within 30 days. Major complications consisted of the Clavien-Dindo IV (CDIV) complications or higher.^15^ The primary predictor of complications was reported history of disseminated or metastatic cancer. Patients were then divided into two groups, including (1) patients with a diagnosis of disseminated cancer (Met) and (2) patients without a diagnosis of disseminated cancer (Non-Met). Indication for surgery was identified by ICD-10 code and included: prosthetic joint infection (PJI), oncologic, patellar pain/instability, joint instability, stiffness/contracture, dislocation, periprosthetic fracture, pain (undefined), pathologic fracture, osteolysis, implant complication (loosening or failure), or polyethylene wear. It should be noted that patients may have undergone revision surgery for multiple indications and, therefore, there may be overlap present amongst these populations.

### Data Analyses

Baseline demographic variables and surgical indications were reported as either prevalence (%) or median with interquartile range (IQR). 30-day outcome variables were reported as incidence (%). Initial unadjusted student’s t-test was utilized to compare outcome variables between Met and Non-Met patients. Multivariate analysis (either logistic regression or ANCOVA) controlling for statistically significant baseline confounders was performed to compare associations between Met patients and outcomes within both revision TKA and THA. The effect sizes were reported as odds ratio (OR) and 95% confidence interval (95% CI). Further multivariate analysis was utilized to compare the associations with outcomes between Met patients and RAI-rev Severely Frail patients within both revision TKA and THA. The associations with outcomes were compared between Met revision TKA patients and Met revision THA patients using multivariate analysis. Discriminatory accuracy of each preoperative index (RAI-rev, mFI-5, age, ASA) was assessed using Receiver Operating Characteristic (ROC) analysis with area under the curve/C-statistic generated for each model. The discriminatory accuracy of the frailty models was compared using the DeLong test. In all testing, significance was established a-priori for odds ratios and 95% confidence intervals exclusive of 1.0 and *P* < .05. All statistical analyses were conducted using SPSS, version 29.1.1 (Armonk, NY).

## Results

Question 1: Do patients with history of metastasis undergo revision TJA for the same indications as unaffected peers?

Indications for surgery for Met and Non-Met in revision TKA and THA groups were highlighted in Table 2. Met patients had higher rates of undergoing revision TKA for PJI and periprosthetic fracture but lower rates for implant-related complications. Met patients had higher rates of undergoing revision THA for PJI and dislocation and lower rates for implanted-related complications.

**Table 2.** Surgical Indications

Indication	Met rTKA (%)	Non-met rTKA (%)	*P*	Met rTHA (%)	Non-met rTHA (%)	*P*
Prosthetic joint infection	40.7	16.5	<.001	18.7	12.4	.026
Oncologic	2.5	0.1	<.001	7.0	0.1	<.001
Patellar pain/instability	1.2	0.4	.195	—	—	—
Joint instability	7.4	12.6	.157	0.7	0.7	.938
Dislocation	—	—	—	26.1	19.4	.045
Stiffness/contracture	1.2	1.4	.891	0.0	0.3	.539
Pain	3.7	10.3	.052	5.6	6.7	.625
Periprosthetic fracture	7.4	3.1	.023	11.3	11.1	.961
Pathologic fracture	1.2	0.1	<.001	7.0	0.1	<.001
Osteolysis	1.2	0.7	.558	0.7	3.2	.091
Implant complication	28.4	43.7	.006	15.5	36.8	<.001
Implant loosening	9.9	24.2	.003	7.0	17.5	.001
Implant failure	4.9	3.5	.495	2.8	4.4	.368
Articular bearing surface wear	0.0	2.9	.189	1.4	4.5	.055

Question 2: Are patients with history of metastasis at higher risk for complications following revision total joint arthroplasty compared to patients without metastasis?

### 30-Day Outcomes for Revision TKA

The unadjusted 30-day outcome comparison for Met and Non-Met patients undergoing revision TKA are displayed in Table 3. After controlling for baseline confounders, Met patients were more likely to experience any complication (OR: 2.56, CI: [1.48–4.43]), major complication (OR: 2.17, 95% CI: [1.24–3.82]), and mortality (OR: 7.99, 95% CI: [2.70–23.65]), as well as extended length of stay (>3 days), need for postoperative blood transfusion, pulmonary complications, and sepsis (all *P* < .01). When examining comparing Met patients to RAI-rev Severely Frail patients, multivariate analysis demonstrated Met patients were more likely to experience pulmonary complications (OR: 3.45, 95% CI: [1.05–11.31]) and mortality (OR: 4.86, 95% CI: [1.52–15.48]).

**Table 3.** 30-Day Outcomes for Revision TKA

Surgery	Met (%)	Non-met (%)	*P*-value
Any complication	24.7	10.0	<.001
Major (Clavien-Dindo 3+)	22.2	8.8	<.001
Surgical site infection	1.2	0.6	.474
Urinary tract infection	2.5	0.6	.030
Sepsis	7.4	1.5	<.001
Cardiac	0.0	0.4	.549
Need for blood transfusion	24.7	6.5	<.001
Pulmonary	6.2	0.7	<.001
Deep vein thrombosis	0.0	0.6	.469
Pulmonary embolism	1.2	0.4	.209
Mortality	6.2	0.3	<.001
Extended length of stay	63.0	25.2	<.001
Readmission	9.9	5.5	.086
Reoperation	4.9	3.4	.435

### 30-Day Outcomes for Revision THA

The unadjusted 30-day outcome comparison for Met and Non-Met patients undergoing revision THA are displayed in Table 4. After controlling for baseline confounders, Met patients were more likely to experience any complication (OR: 2.40, CI: [1.65–3.49]), major complication (OR: 2.19, 95% CI: [1.47–3.25]), DVT (OR: 4.82, 95% CI: [1.92–12.10]), and mortality (OR: 3.67, 95% CI: [1.43–9.41]), as well as extended length of stay (>3 days), need for postoperative blood transfusion, cardiac complications, and sepsis (all *P* < .01). When comparing Met patients to RAI-rev Severely Frail patients, multivariate analysis demonstrated Met patients were not more likely to experience any complication.

**Table 4.** 30-Day Outcomes for Revision THA

Surgery	Met (%)	Non-met (%)	*P*-value
Any complication	30.3	14.5	<.001
Major (Clavien-Dindo 3+)	24.7	12.4	<.001
Surgical site infection	0.7	0.9	.782
Urinary tract infection	0.7	1.3	.498
Sepsis	4.9	1.7	.003
Cardiac	3.5	0.8	<.001
Need for blood transfusion	36.6	21.6	<.001
Pulmonary	2.1	1.2	.334
Deep vein thrombosis	3.5	0.7	<.001
Pulmonary embolism	1.4	0.4	.088
Mortality	3.5	0.8	<.001
Extended length of stay	63.4	40.1	<.001
Readmission	13.4	8.2	.024
Reoperation	7.0	6.2	.672

Question 3: Is there a higher risk for complications during revision TKA or revision THA in patients with metastatic cancer?

### 30-Day Outcomes for Met Patients Undergoing Revision TJA

The unadjusted 30-day outcome comparison for Met patients undergoing revision THA vs TKA are displayed in Table 5. After controlling for baseline confounders, Met patients were more likely to experience any complication (OR: 2.40, CI: [1.65–3.49]), major complication (OR: 2.19, 95% CI: [1.47–3.25]), DVT (OR: 4.82, 95% CI: [1.92–12.10]), and mortality (OR: 3.67, 95% CI: [1.43–9.41]), as well as extended length of stay (>3 days), postoperative blood transfusions, cardiac complications, and sepsis (all *P* < .01). When comparing Met patients to RAI-rev Severely Frail patients, multivariate analysis demonstrated Met patients were not more likely to experience any complication.

**Table 5.** 30-Day Outcomes for Met Revision THA vs TKA

Surgery	Met THA (%)	Met TKA (%)	*P*-value
Any complication	30.3	24.7	.375
Major (Clavien-Dindo 3+)	24.7	22.2	.684
Surgical site infection	0.7	1.2	.688
Urinary tract infection	0.7	2.5	.273
Sepsis	4.9	7.4	.450
Cardiac	3.5	0.0	.088
Need for blood transfusion	36.6	24.7	.067
Pulmonary	2.1	6.2	.118
Deep vein thrombosis	3.5	0.0	.088
Pulmonary embolism	1.4	1.2	.914
Mortality	3.5	6.2	.360
Extended length of stay	63.4	63.0	.951
Readmission	13.4	9.9	.443
Reoperation	7.0	4.9	.535

Question 4: Is frailty more predictive of outcomes following revision TJA?

### Predictability of Overall 30-Day Outcomes by Indices

The predictability of overall 30-day outcomes in revision total joint arthroplasty by the RAI-rev, mFI-5, ASA classification, and age is displayed in Table 6. The RAI-rev had the highest AUC for all outcomes except for any complication and non-home discharge. The RAI-rev had superior predictability for extended length of stay (0.625 [0.619–0.630]) and mortality (0.851 [0.824–0.880]) compared to all other indices.

**Table 6.** Predictability of 30-Day Outcomes

Index	RAI-rev	mFI-5	Age	ASA
Any complication	0.588 [0.579–0.597]	0.571 [0.563–0.579]	0.556 [0.548–0.565]	0.591 [0.583–0.599]
Pulmonary	0.703 [0.676–0.730]	0.658 [0.632–0.685]	0.639 [0.611–0.667]	0.670 [0.644–0.696]
Cardiac	0.747 [0.717–0.776]	0.645 [0.613–0.678]	0.715 [0.683–0.747]	0.663 [0.630–0.695]
Deep vein thrombosis	0.589 [0.555–0.622]	0.547 [0.513–0.580]	0.567 [0.533–0.600]	0.564 [0.533–0.596]
Mortality	0.851 [0.824–0.880]	0.701 [0.665–0.737]	0.802 [0.783–0.821]	0.735 [0.703–0.767]
Non-home discharge	0.694 [0.688–0.700]	0.600 [0.594–0.606]	0.698 [0.692–0.704]	0.629 [0.624–0.635]
Extended length of stay	0.625 [0.619–0.630]	0.571 [0.565–0.577]	0.600 [0.594–0.606]	0.609 [0.604–0.615]

## Discussion

Cancer remains a significant healthcare challenge, being the second-leading cause of mortality in the United States and responsible for one in every four deaths.^16^ Despite the stabilization of incidence rates in the U.S., global rates continue to rise, the number of cancer survivors has also increased in addition to their quality of life, leading to more cancer survivors undergoing primary and revision arthroplasty.^8,17-20^ Our study found patients with a diagnosis of disseminated cancer undergoing both revision TKA or THA are at a significantly higher risk of all complications, especially mortality compared to their non-affected peers. These patients are more often undergoing revision for infectious indications in both TKA and THA, periprosthetic fracture in TKA, and dislocation-related complications in THA. While these procedures may have an elevated risk in this population, frailty as assessed by the revised Risk Analysis Index (RAI-rev) may be a relatively reliable predictor of outcomes of revision arthroplasty compared to other indices and may provide better understanding of this risk and direction of preoperative optimization strategies.

Our findings are consistent with previous reports that there is an increased risk in mortality after revision TKA in patients with increasing age, greater comorbidity burden, underweight or normal BMI, insulin-dependent diabetes, septic revision, and general anesthesia, all of which were seen in our metastatic cohort.^21^ As predicted, these patients also experienced greater postoperative complications, including major complications. One previous study highlighted patients with a history of disseminated cancer may have significantly increased rates of complications when undergoing primary TJA.^1^ Similarly, cardiac comorbidities have been found to play a major role in postoperative complications in revision THA, especially in blood loss, transfusions, ICU stays, length of stay, re-revisions, and duration of surgery.^22^ The higher rates of CHF within our Met cohort may have further contributed to their surgical risk. In addition, Karam et al found patients with a history of metastatic cancer undergoing THA to have higher rates of DVT, cardiac complications, and mortality, all of which were validated in our study.^23^

As of 2024, there are about 623 405 individuals living in the United States with disseminated cancer, including prostate, bladder, colorectal, breast, lung, or melanoma, with an expected >10% increase by the year 2025.^24^ While radiation therapy has been theorized to increase the rates of component loosening and subsequent implant failure or periprosthetic fracture, our study found a lower proportion of Met patients undergoing revision for these indications compared to Non-Met patients.^25^ Although previous meta-analyses have sought to validate this proposed risk, the studies reviewed were largely low-level data without specific details on implant material or positioning.^26,27^ Likewise, this has been recently studied with no association found between hip implant loosening and undergoing radiation treatment.^28^ However, as evidenced by Houdek et al, the incidence of dislocation-related complications for patients with an oncological diagnosis was significantly higher in this cohort.^29^ It can also be understood that patients with metastatic disease may be at risk for PJI given the metabolic abnormalities and immunosuppressive medications undertaken, however the invasiveness required during the primary procedure could not be accounted for and may confound the adequate risk appropriated to history of metastasis.^30^

These findings, however, are reporting on a heterogenous cohort of patients with history of metastatic disease. These patients were not stratified based on lesion grading or primary etiology, which significantly limits the direct clinical applicability of our findings to inform physicians. Yet, these results should alert surgeons of the potential risks for a patient with any history of metastatic disease to further investigate their current stage and treatment, and critically attempt to optimize their care to minimize possible risk of perioperative complications.

Given the complications of rTJA, especially in patients with a history of disseminated cancer, it is critical for orthopaedic surgeons to consider the risk-benefit analysis and discuss possible outcomes with their patients. Identifying preoperative risk factors can help better understand potential complications allowing surgeons and hospitals to be prepared with resources and patient education. While our study highlighted the increased surgical risk of revision arthroplasty in this cohort, we also identified relevant predictors for certain complications following revision TJA. In doing so, we found the RAI-rev to have the highest C-statistic for all but two outcomes measured, with a statistically significantly higher predictability for extended length of stay (>3 days) and mortality. Although the differences were not statistically significant across all outcomes, these findings may be more clinically relevant, as a physician will only need to calculate the score once to provide informed risk for all complications compared to age or ASA. Although the RAI-rev has not been previously studied within arthroplasty, the association with outcomes mimic those found in spine and plastic surgery and may provide further recommendation for its use across orthopaedic surgery, especially in high-risk contexts.^14,31-33^

This study has several limitations. First, it is a retrospective observational study which does not allow for definitive causality. Secondly, like any database, NSQIP has the potential for diagnosis and procedure coding errors impacting the quality of data and possible missing data. Not all hospitals in the U.S are required to participate in data entry for NSQIP which allows limited data analysis. Surgical approach, implant company, alignment, radiographic variables, technique and number of previous procedures could not be elucidated within the current dataset and may impact the direct clinical translatability of these findings. Additionally, NSQIP does not differentiate the specific types of disseminated cancer and limits findings reported within the 30-day postoperative period which can impact clinical prognosis and outcomes. The Met cohort likely represents a heterogenous cohort with some having undergone a primary TJA for oncologic indications, pelvic radiation for unrelated oncologic disease, or have a remote history of metastasis that no longer affects their current risk profile. We were not also able to determine the acuity of metastatic cancer, current/past therapeutic treatment, and prognosis related to the cancer, which may have significant impact on their ability to undergo revision and their outcome. We are unable to differentiate type of prosthesis, implant company, technique, and whether a megaprosthesis was used based on CPT code, which may affect the outcome of the revision surgery and place patients at differing risks following the procedures. Previous studies have noted the increased use of megaprostheses in this population associated with increased rates of complications, including infection, revision, and mortality, due to increased soft tissue dissection and bone loss, which may play as a significant confounder in the results of our analysis.^34,35^ A key limitation is the grouping of all patients with metastatic cancer into a single category, without accounting for differences in cancer type, extent of disease, multiple indications for revision that were not recorded in the database, or systematic treatments. Furthermore, restricting the analysis to a 30-day postoperative period limits evaluation of critical outcomes in patients with metastatic disease which includes implant longevity, cancer progression, and delayed complications. Finally, there are unmeasured confounding limitations and factors that could not be accounted for within the present dataset and can impact data analysis and associations.

## Conclusion

Patients with metastatic cancer have significantly elevated risk of complications following revision joint arthroplasty but may have moderate predictability by frailty assessment. The predicted increased demand for revision arthroplasty precipitates the need for a better understanding of the risks and complications patients with metastatic disease may encounter when undergoing revision arthroplasty to allow surgeons to better educate patients. Surgeons can utilize this information to emphasize protective strategies to mitigate risk during and following total joint arthroplasty.

## Data Availability

Data utilized for this study is readily available upon request.*
